# Dose–response of epidural ropivacaine with 0.4 μg mL^‐1^ of dexmedetomidine for labor analgesia: A prospective double-blinded study

**DOI:** 10.1097/MD.0000000000039654

**Published:** 2024-09-20

**Authors:** Jun Yin, Shen Cao, Jie Lei, Xiao-Yan Wang, Jing-Ping You, Ding-Chao Xu, Xin-De Chen, Wen-Ping Xu

**Affiliations:** a Department of Anesthesia, Women and Children Hospital of Jiashan, Jiaxing, China; b Department of Anesthesia, The First People’s Hospital of Jiashan, Jiaxing, China; c Department of Anesthesia, The First People’s Hospital of Pinghu, Zhejiang, China.

**Keywords:** dexmedetomidine, dose–response, epidural, labor analgesia, ropivacaine

## Abstract

**Background:**

Studies have shown that the ideal dose of epidural dexmedetomidine is 0.4 μg mL^‐1^ for epidural labor analgesia. However, the appropriate dose of ropivacaine when combined with 0.4 μg mL^-1^ of dexmedetomidine for epidural labor analgesia is still unknown. Therefore, we aimed to determine the dose–response of ropivacaine when using 0.4 μg mL^-1^ of dexmedetomidine as epidural adjuvant for labor analgesia.

**Methods:**

One hundred of nulliparous singleton pregnant patients were randomized allocated into 1 of 5 groups with epidural ropivacaine concentration of 0.05%, 0.0625%, 0.075%, 0.0875%, and 0.1%. Labor analgesia was initialed with 12 mL of the mixed study solution. Effective analgesia was defined as a visual analogue scale <10 mm 30 minutes after the initial epidural bolus. The EC50 and EC95 for epidural ropivacaine was calculated by probit regression.

**Results:**

Ninety-three of parturients were involved into the final analysis. Totals of 63.2% (12/19), 73.7% (14/19), 88.9% (16/18), 94.7% (18/19), and 100% (18/18) of parturients in group 0.05, 0.0625, 0.075, 0.0875, and 0.1 received effective epidural labor analgesia. The calculated EC50 and EC95 of epidural ropivacaine were 0.046% (95% CI 0.028–0.054%) and 0.086% (95% CI 0.074–0.137%), respectively.

**Conclusions:**

Under the condition of the study, a bolus of 12 mL ropivacaine 0.086% and dexmedetomidine 0.4 μg mL^‐1^ could afford 95% of nulliparous singleton pregnant patients without suffering labor pain after a test dose of lidocaine 45 mg.

## 1. Introduction

Recently, “low concentration and high volume of local agent” technique has been advocated in epidural labor analgesia (ELA), with the purpose of reducing the incidence of side effects of epidural block, such as hypotension and lower limbs motor block.^[1–3]^ Epidural adjuvants are commonly used to reduce the concentration of epidural local anesthetics.^[4–7]^ Opioids, such as fentanyl and sufentanil are usually used as adjuvants for ELA, but associated with some undesired side effects,^[8,9]^ thus non-opioid adjuvants are becoming important.

Dexmedetomidine has been studied in ELA and found it was an effective epidural adjuvant for labor analgesia.^[6,8,10–12]^ Moreover, Liu et al found the ideal dose of dexmedetomidine for ELA is 0.4 μg mL^‐1^ using an up-and-down sequential allocation to determined the median effective of epidural ropivacaine.^[8]^ However, the dose–response of ropivacaine with dexmedetomidine is still remains unknown, when using dexmedetomidine 0.4 μg mL^‐1^ as epidural adjuvant. To determine the ideal dose of ropivacaine is appropriate for minimizing the side effects of epidural block such as motor block and hypotension. Therefore, we conducted a prospective, randomized and double-blinded study to determine the EC50 and EC95 for ropivacaine when combined with dexmedetomidine 0.4 μg mL^‐1^ for ELA.

## 2. Patients and methods

### 2.1. Design

This study was approved by the Ethical Committee of Women and children Hospital of Jiashan on July 28th, 2021 (KY2021-037). All patients involved in this study signed the written informed consent. We registered this clinical trial in the Chinese Clinical Trial Registry on June 15th, 2022 (registry number ChiCTR2200061040).

This study started clinical trials on June 20, 2022 and took 1 year to complete.

### 2.2. Subjects and setting

Inclusion criterions for this study were as follows: healthy singleton pregnancies, gestational age ≥ 37 weeks, American Society of Anesthesiologists Physical Status II, spontaneous onset of labor, latent phase of labor with cervical dilation of 2 to 5 cm, and painful contractions requiring labor epidural analgesia. Exclusion criterions were defined as: preeclampsia or hypertension, preexisting or gestational diabetes, BMI > 35 kg/m^2^, any contraindication to regional anesthesia, allergy or hypersensitivity to ropivacaine or dexmedetomidine, or if they had received sedatives within 4 hours preceding epidural insertion.

### 2.3. Study protocol

One hundred of nulliparous singleton pregnant patients were randomized allocated into 1 of 5 groups with epidural ropivacaine concentration of 0.05%, 0.0625%, 0.075%, 0.0875%, and 0.1%. The randomization scheme was prepared by Jun Ying, who was not involved in patients’ pain management and data collection, in advance of patients’ enrollment by using Microsoft Excel (Microsoft Corporation, Redmond, WA). Then the randomized scheme was kept in sequentially numbered opaque envelopes and opened after the first patient enrollment. Ropivacaine and dexmedetomidine 0.4 μg/mL was prepared in advance in a sterilized condition by a fixed anesthesia assistant who did not involve in patients management. All study participants were blinded to their assignment.

Upon the patient arriving in labor room, an 18-G of venous indwelling needle was used to establish a peripheral venous access in the left upper arm. A standard monitoring including noninvasive blood pressure cuff, pulse oximeter, electrocardiography leads (including respiratory rate monitor), and fetal heart rate monitor was applied. The baseline systolic pressure and heart rate that defined as the mean of 3 readings between uterine contraction intervals were recorded. An infusion of 250 mL of lactated Ringer solution was initiated.

Then the epidural technique was accomplished. In brief, the patient was positioned in a left lateral position, the epidural space was determined at the L3–4 interspace via ultrasound assessment by losing of resistance to saline (<2 mL) technique; then a epidural multiport wire-reinforced flexible catheter (19G; Zhejiang Runqiang Medical Instruments Co. Ltd., Jiaxing, Zhejiang, China) was then inserted 4 to 5 cm into the epidural space by 1 of 4 attending anesthesiologists (Shen Cao, Jie Lei, Xiao-Yan Wang, Ding-Chao Xu). As a test dose after gently aspirated the catheter, a combination of 3 mL lidocaine 1.5% and 15 μg epinephrine was injected through the catheter.

If no signs of subarachnoid and intravenous injection were detected after 5 minutes, 12 mL of study solution with dexmedetomidine 0.4 μg/mL was administered via epidural catheter as an inducing bolus to relieve the labor pain over 60 seconds. The solution was prepared by Jun Yin who is aware of patients’ grouping in a sterile condition. The parturients received 0.05%, 0.0625%, 0.075%, 0.0875%, and 0.1% of ropivacaine according to patient assignment. Thirty minutes after the inducing bolus, parturients were asked to report their pain on a standard visual analogue scale (VAS) of 0 to 100.^[13]^ When a parturient reported a VAS score <10, it was regarded as an effective analgesia, otherwise it was regarded as an ineffective analgesia. If there was an ineffective analgesia, 10 mL of 1% lidocaine was given via epidural catheter to supplement the analgesia. If the patient still reported a VAS score > 10, it was regarded as a failed epidural catheter, and this case was excluded from the study. The study was terminated after 30 minutes of the inducing bolus.

### 2.4. Measurements

The primary outcome of this study was effective or ineffective analgesia, which were defined a VAS score was less or more than 10 mm after 30 minutes of the inducing epidural bolus. Secondary outcomes including sensory block level, motor block (evaluated via the Bromage scale,^[14]^ where 0 = could move all joints in the leg, 1 = could bend the knees and ankles, 2 = could only move the ankle, and 3 = could not move the leg at any joint), side effects (hypotension defined as the SBP < 90 mm Hg or <80% of baseline SBP, and treated with intravenous phenylephrine at a dose of 50 mcg; maternal bradycardia defined as the HR < 60 bpm, fetal bradycardia defined as a heart rate < 110 bpm, respiratory depression defined as oxygen saturation < 90%), neonatal outcomes (neonatal weight, 1 minute and 5 minutes Apgar score, pH value and lactic acid of umbilical artery blood) were also recorded. Patient satisfaction of labor analgesia, which was ranked as 1 (not satisfied at all) to 10 (fully satisfied) verbal score, was investigated 30 minutes after the inducing bolus. Supplementary analgesia was initialed with epidural ropivacaine 0.1% according to our institutional standard practice at the anesthesiologists’ discretion when necessarily.

### 2.5. Sample size calculation and statistical analysis

We calculated the sample size via the Cochran-Armitage Test by using Power Analysis and Sample Size (version 11.0.7; NCSS, LLC, Kaysville, UT). According to our preliminary data for the 5 groups with the ropivacaine concentration of 0.05%, 0.0625%, 0.075%, 0.0875%, and 0.1% 12 mL (equals to 6.0, 7.5, 9.0, 10.5, and 12 mg) the proportions of effective analgesia were 40%, 55%, 80%, 90%, and 95%, respectively. A sample size of 10 patients for each group (50 patients in total) are needed with a 90% power to detect a linear trend in the proportion of patients with effective pain regimen among groups by using a Z test with continuity correction and a significance level of 0.05. Taken account for possible dropouts, the sample size was increased to 20 patients for each group.

The continuous variables were checked for normally or non-normally distributed using graphical displays of the data and the Kolmogorov–Smirnov test. Normally distributed data was presented as mean standard deviation and tested for significance via one-way analysis of variance with post-Bonferroni tests for pairwise comparisons, meanwhile the non-normally distributed data was presented as median [range] and tested with the Kruskal–Wallis test, and the post Dunns test was used to test pairwise comparisons. For categorical data such as the incidence of side effects, we used the Cochran–Armitage χ2 test for trend; if the overall test was significantly different among groups, the χ2 tests were applied for pairwise comparisons. The median effective concentration (EC50) and 90% effective concentration (EC95) of epidural ropivacaine was calculated by Probit regression. The null hypothesis of the adequacy of the probit model fit to the data was tested using the chi-square test for goodness-of-fit, as determined by Pearson. A *P* value < .05 was regarded as statistically significant (two-sided). Analyses were performed using IBM SPSS Statistics for Windows version 22.0 (IBM Corp, Armonk, NY) and GraphPad Prism version 5.0 (GraphPad Software Inc., San Diego, CA).

## 3. Results

**Figure 1.**
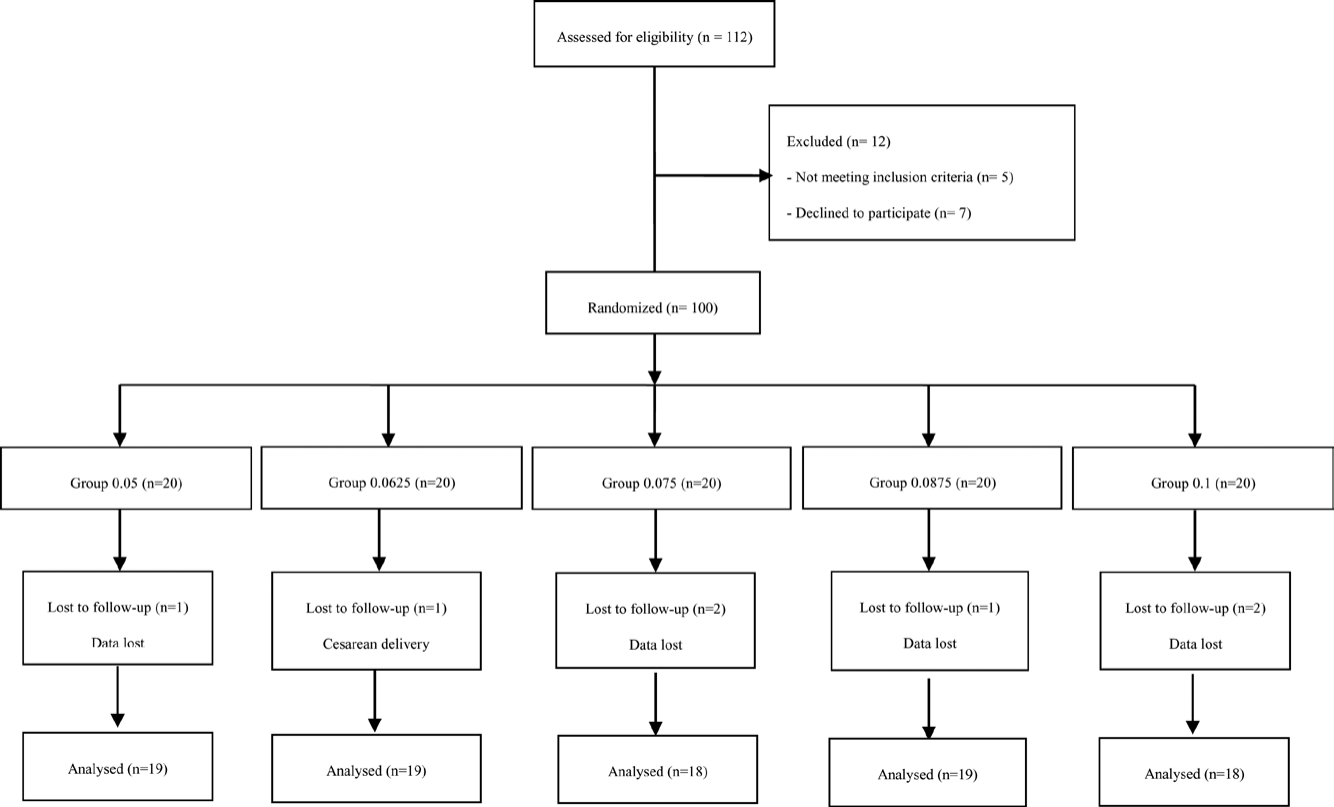
CONSORT diagram.

Of the initial 100 participants enrolled with written informed consent, 7 patients were excluded for the failed epidural catheter and transferred for cesarean delivery. Eventually, data from 93 participants were involved in the final analysis (Fig. 1). Patient demographic data are shown in Table 1.

**Table 1:** Parturient characteristics.

	Group 0.05 (n = 19)	Group 0.625 (n = 19)	Group 0.75 (n = 18)	Group 0.875 (n = 19)	Group 0.1 (n = 18)	*P* value
Age (years)	29.3 (4.5)	28.2 (3.6)	28.3 (2.8)	29.2 (4.2)	26.3 (3.5)	.14
BMI (kg m^‐2^)	27.6 (3.6)	27.4 (3.8)	28.5 (3.2)	26.0 (2.4)	26.0 (3.4)	.08
Gestational age (weeks)	39.6 (1.1)	39.2 (1.2)	39.5 (1.0)	39.8 (0.9)	39.8 (1.2)	.31
Pain score at request for epidural analgesia (VAS 0–100 mm)	76 (6)	76 (7)	75 (9)	77 (8)	76 (7)	.91
Cervical dilation at request for epidural analgesia (cm)	2 (2-2 [2-3])	2 (2-2 [2-3])	2 (2-2 [2-3])	2 (2-2 [2-3])	2 (2-2 [2-3])	.86

Data are presented as mean (SD) or median (IQR [range]) as appropriate.

BMI = body mass index, IQR = interquartile range, SD = standard deviation, VAS = visual analog score.

There were 7, 5, 2, 1, and no patients in each group need supplementary bolus of rescued analgesia 30 minutes after the inducing epidural bolus. There was a significant liner trend across groups, *P* < .001. There were more patients in group 0.05 and group 0.0625 required additional rescued analgesia 30 minutes after the inducing bolus for the labor pain than in the other groups, *P* < .05.

**Figure 2.**
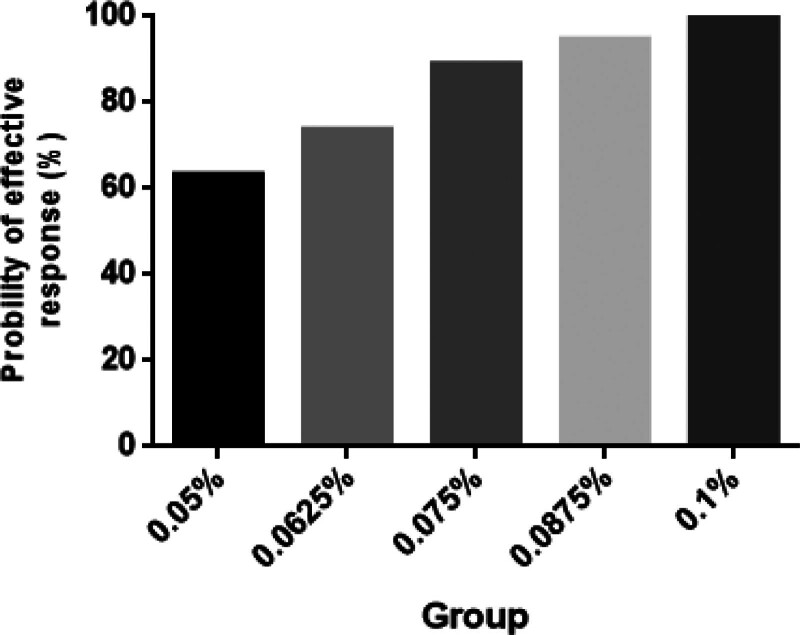
Proportion of patients with effective induction of analgesia at different concentration of ropivacaine.

**Figure 3.**
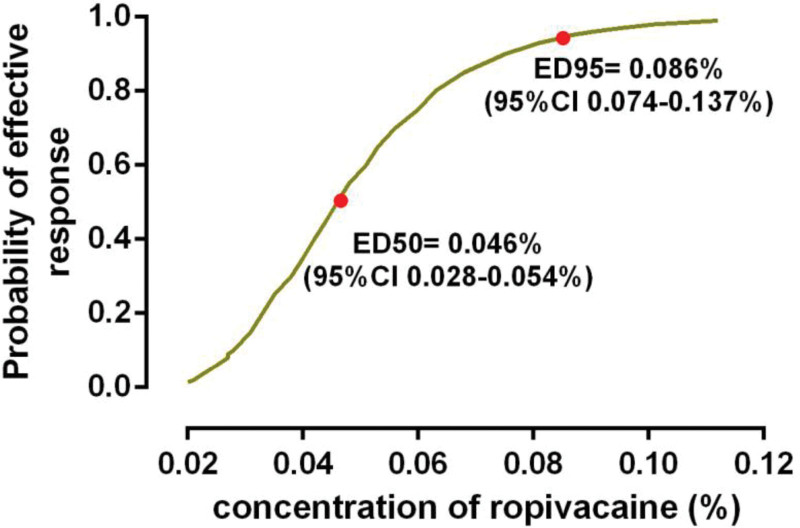
Dose–response curve of ropivacaine at corresponding concentration (%) for induction of epidural labor analgesia. The EC50 and EC95 values were 0.046% (95% CI 0.028–0.054%) and 0.086% (95% CI 0.074–0.137%), respectively.

The incidence of effective labor analgesia was 63.2%, 73.7%, 88.9%, 94.7%, and 100% in group 0.05, 0.0625, 0.075, 0.0875, and 0.1, respectively (Fig. 2). The dose–response curve of ropivacaine with dexmedetomidine 0.4 μg/mL under epidural analgesia for inducing labor analgesia was shown in Figure 3. The EC50 and EC95 values were 0.046% (95% CI 0.028–0.054%) and 0.086% (95% CI 0.074–0.137%), respectively. The results of the Pearson goodness-of-fit chi-square test suggested a satisfactory fit of the probit model (*P* = .547).

**Figure 4.**
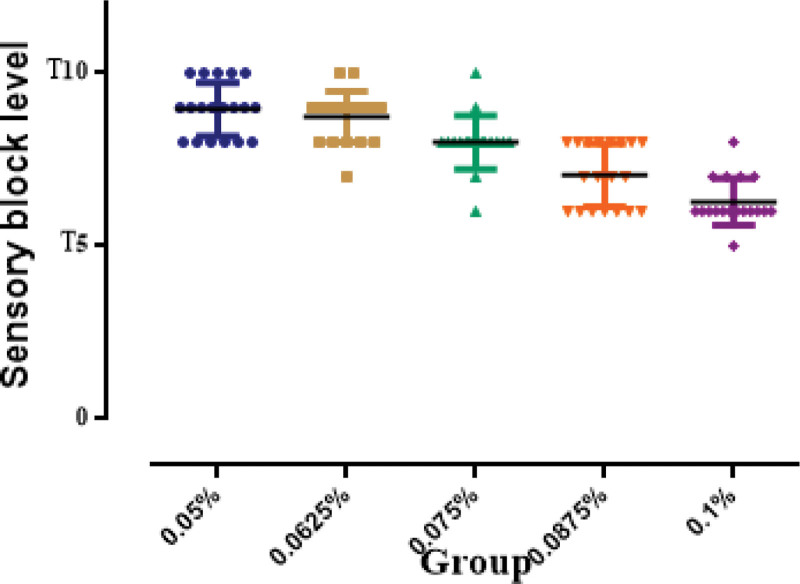
The highest sensory block level among groups. Black lines show median and error bars show interquartile range.

There were more patients in group 0.0875 and 0.1 developed a higher sensory block to alcohol wipes comparing with other groups. The highest sensory block level among groups was shown in Figure 4. There was 1 patient in group 0.075 and 1 patient in group 0.1 developed hypotension and no patient required vasopressor therapy for hypotension. There was no patient developed motor block in lower limb. The characteristic of epidural analgesia and side effects were shown in Table 2.

**Table 2:** Secondary outcomes.

	Group 0.05 (n = 19)	Group 0.625 (n = 19)	Group 0.75 (n = 18)	Group 0.875 (n = 19)	Group 0.1 (n = 18)	*P* value
Duration of first labor stage (minutes)	357 (138)	444 (188)	442 (222)	456 (150)	447 (195)	.43
Duration of second labor stage (minutes)	38 (14)	22 (22)	44 (23)	39 (15)	45 (18)	.65
Operative vaginal delivery	1 (5.3)	2 (10.5)	0 (0)	1 (5.3)	1 (5.5)	.77
Neonatal weight	3166 (410)	3314 (373)	3217 (258)	3214 (228)	3228 (210)	.66
Hypotension	0 (0)	0 (0)	1 (5.5)	0 (0)	1 (5.5)	.30
Nausea/vomiting	3 (15.8)	5 (26.3)	3 (16.7)	4 (21.0)	3 (16.7)	.91
Patient satisfaction	3 (2–3)	3 (3–4)	3 (3–4.25)	4 (3–5)*	5 (5–5)*	<.001
1-minute Apgar score	10 (9–10)	10 (8–10)	10 (8–10)	10 (7–10)	10 (8–10)	.33
5-minute Apgar score	10 (9–10)	10 (8–10)	10 (9–10)	10 (8–10)	10 (9–10)	.63
pH of umbilical artery blood	7.31 (0.06)	7.31 (0.05)	7.30 (0.06)	7.30 (0.06)	7.32 (0.07)	.23
Lactic acid of umbilical artery blood	4.31 (0.37)	4.05 (0.46)	4.39 (0.43)	4.31 (0.36)	4.30 (0.34)	.40

Data are presented as mean (SD), median (IQR [range]) or number (%) as appropriate as appropriate.

IQR = interquartile range, SD = standard deviation.

* Adjusted *P* < .05 when compared to group 0.05, 0.0625, and 0.75 using post hoc Dunn test.

The outcomes of neonatal and patient satisfaction were also presented in Table 2. There were no significant differences in neonatal weight, 1 minute and 5 minutes Apgar score, pH value and lactic acid of umbilical artery blood among groups. Patients in group 0.875 and 0.1 acquired a similar median satisfaction of 95 verbal score, which was higher than in other groups.

## 4. Discussion

Ropivacaine with dexmedetomidine have been investigated and deemed as effective and safe combinations for ELA. Although dose-response of studies have been reported for epidural dexmedetomidine for labor analgesia, limited data are available for the ideal epidural ropivacaine concentration or dose. In this prospective, randomized dose allocation study, we observed 12 mL of 5 different concentration of ropivacaine (doses range from 6 to 12 mg). Use this range, we calculated the EC50 and EC95 of epidural ropivacaine were 0.046% (95% CI 0.028–0.054%) and 0.086% (95% CI 0.074–0.137%), respectively, when combined with dexmedetomidine 0.4 μg/mL. Results of this study should be interpreted, 12 mL of ropivacaine 0.046% (EC50) in this study equals to the median effective dose (ED50) that a dose of 5.5 mg (95% CI 3.4–6.5 mg), which would provide effective analgesia in 50% patients; similarly 12 mL of ropivacaine 0.086% (EC95) equals to the 95% effective dose (ED95) that a dose of 10.3 mg (95% CI 8.9–16.4 mg) which would afford 95% patients without labor pain under the study condition in early labor.

Prior studies in the context of epidural ropivacaine using different methodologies for labor analgesia have been reported. Zhang et al^[10]^ determined the EC50 of epidural ropivacaine combined with dexmedetomidine 0.5 μg/mL using the up-and-down sequential allocation for labor analgesia and found the EC50 was 0.062% and with a 95% CI range from 0.058% to 0.066%. Xiang et al^[15]^ reported a clinical trial comparing the EC50 of ropivacaine whether using sufentanil 0.5 µg/mL as epidural adjuvant and found the EC50 was decreased from 0.109% (95% CI, 0.105–0.112%) to 0.085% (95% CI, 0.079–0.090%). In comparison, our results for EC50 of 0.046% (95% CI 0.028–0.054%) was significant lower than that reported in these studies. However, Liu et al^[8]^ estimated the EC50 of epidural ropivacaine with different concentration of dexmedetomidine using up-and-down sequential allocation. They found the ideal concentration of dexmedetomidine was 0.4 μg/mL and accordingly the concentration of epidural ropivacaine was 0.044% (95% CI, 0.036–0.045%), which was similar with the current results. The following factors that can account for the different results among studies.

Firstly, different methods for study protocol and statistical analysis could result in inconsistent results. For example, we used a traditional random allocation dose-finding methodology and probit analysis to determine the relationship between different dose and effective analgesia. Otherwise, in comparison, Zhang et al,^[10]^ Xiang et al,^[15]^ and Liu et al^[8]^ used the up-and-down and Massey formula or improved Dixon method to calculate the EC50 only. Moreover, among these studies, different volume of boluses were administered via epidural catheter. In our study, we gave a volume of 12 ml according to our clinical routine. Zhang et al^[10]^ gave the dose in a volume of 10 ml, while Xiang et al^[15]^ used 8 mL and Liu et al^[8]^ used 13 mL. These differences in the volume of epidural local anesthetics may lead to inconsistency results among these studies, although the exact effect is dubious, because of literatures regarding of the dose, concentration, and volume on the spread of epidural local anesthetics remains conflicted. We believed the sensory block level was positive related to the concentration and dose of epidural ropivacaine, when the volume was kept constant, as more patients developed higher block level in group 0.0875 and 0.1 in the current study, which was consistent with previous studies.^[16,17]^

Secondly, different definition of effective analgesia may also lead to different results among studies. In the current study, we defined the effective analgesia as the VAS pain score of <10 (0 to 100) 30 minutes after the injection of the inducing bolus. While, comparing with Zhang et al,^[10]^ Xiang et al,^[15]^ and Liu et al^[8]^ studies, the effective analgesia was defined as a VAS pain score <3 (0 to 10). Lee et al^[16]^ also conducted a dose–response study, and they defined the effective analgesia as a reduction of VAS pain score during a contraction to 50% or less of baseline within 30 minutes after injection. And they reported a ED50 for epidural ropivacaine of 18.4 mg (95% CI, 13.4–25.4 mg), which was also higher than our value of 5.5 mg. In essence, we believed that the lack of objectivity of VAS pain score, combined with the different definitions of effective analgesia in various studies, will inevitably lead to the inconsistency of research results.

Thirdly, adjuvant of different drugs and doses was used in epidural space may result in different value of EC50 of ropivacaine for labor analgesia. Sufentanil was used as adjuvant in Xiang et al^[15]^ study, while dexmedetomidine 0.5 µg/mL was used in Zhang et al^[10]^ study. In Liu et al^[8]^ study, they observed different dose of dexmedetomidine and found a dose-dependent reduction in the median concentration of epidural ropivacaine for labor analgesia. We used dexmedetomidine 0.4 µg/mL and achieved a similar result of EC50 with Liu et al study (0.046% vs 0.044%). There are other various adjuvants for clinical practice including fentanyl, neostigmine, butorphanol, and clonidine for labor analgesia. Further studies to determine which adjuvant is more suitable for labor analgesia may be of great important and interests.

Finally, there are many other factors that may influence a patient’s requirement for analgesia during labor. In ours, Zhang et al^[10]^ and Xiang et al^[15]^ studies, 45 mg of lidocaine were used as the test dose for checking no signs of subarachnoid or intravenous injection, otherwise in Liu et al^[8]^ study the authors did not use lidocaine, which may also influence the results. Moreover, the size of the cervical dilatation, whether primipara or parity, and whether the use of oxytocin are all factors affecting the analgesic effect of labor and also affects the judgment of the outcome.

In this study, the satisfaction for patients of labor analgesia in group 0.0875 and 0.1 is obviously higher than patients in group 0.05, 0.0625, and 0.075 suggests that using a higher dose of local anesthetics nearing the value of ED95 as a bolus for relieving labor pain during the early stage of labor is superior to a low dose nearing ED50. However, some should be also argued that a higher dose could lead to a higher incidence of high sensory block level and associated side effects, such as hypotension. Fortunately, our results showed that there was only 2 patients experienced hypotension and did not need any vasopressor, although there were more patients developed higher sensory block.

Limitations existed in this study. First, the sample size in the current study was chosen to be sufficient to estimate the primary aim of this study but may not be sufficient for some of the secondary outcomes for which the possibility of statistical error cannot be excluded. Second, because of the strict inclusion criterion, we enrolled only single-pregnant nulliparous patients in early labor. Our findings may not be generalizable to multiparous patients or those in more advanced labor. In addition, due to the termination of the study at 30 minutes after administering the inducing bolus, the precise dose-response relationship of ropivacaine with dexmedetomidine for maintenance of labor analgesia remains uncertain. Therefore, additional studies are warranted in order to address this knowledge gap. Third, although floods of literatures reported the efficiency and safety of dexmedetomidine using as an adjuvant to local anesthetics in peripheral and central never block anesthesia,^[6,8,10–12]^ it has not yet to be advocated for use in neuraxial techniques by the U.S. Food and Drug Administration. Therefore, further large sample size and multicenter studies are needed to verify its safety for using in neuraxial block.

In conclusion, under the condition of the study, a bolus of 12 mL ropivacaine 0.086% and dexmedetomidine 0.4 μg mL^‐1^ could afford 95% of nulliparous singleton pregnant patients without suffering labor pain after a test dose of lidocaine 45 mg during the early labor stage.

## Acknowledgments

The authors would like to thank all the staff at the Department of Laboring Room of Women and children Hospital of Jiashan, Zhejiang, China, for their help in this study.

## Author contributions

**Conceptualization:** Jun Yin.

**Data curation:** Shen Cao, Jie Lei, Xiao-Yan Wang, Jing-Ping You, Ding-Chao Xu, Xin-De Chen.

**Formal analysis:** Shen Cao, Ding-Chao Xu.

**Investigation:** Shen Cao, Jie Lei, Jing-Ping You, Ding-Chao Xu, Xin-De Chen.

**Project administration:** Jun Yin, Wen-Ping Xu.

**Supervision:** Jun Yin, Xiao-Yan Wang, Wen-Ping Xu.

**Writing – review & editing:** Jun Yin, Wen-Ping Xu.

**Writing – original draft:** Shen Cao, Jie Lei, Xiao-Yan Wang, Xin-De Chen, Wen-Ping Xu.

## References

[1] Halpern SH Carvalho B. Patient-controlled epidural analgesia for labor. Anesth Analg. 2009;108:921–8. 19224805 10.1213/ane.0b013e3181951a7f

[2] Gomes ME Balle VR Machado SB Mendes FF. Comparison between 0.125% and 0.25% bupivacaine associated to fentanyl for epidural labor analgesia. Rev Bras Anestesiol. 2004;54:467–72. 19471754 10.1590/s0034-70942004000400002

[3] Pandya ST. Labour analgesia: recent advances. Indian J Anaesth. 2010;54:400–8. 21189877 10.4103/0019-5049.71033 PMC2991649

[4] Nevo A Aptekman B Goren O Matot I Weiniger CF. Labor epidural analgesia onset time and subsequent analgesic requirements: a prospective observational single-center cohort study. Int J Obstet Anesth. 2019;40:39–44. 31230990 10.1016/j.ijoa.2019.05.008

[5] Lee AI McCarthy RJ Toledo P Jones MJ White N Wong CA. Epidural labor analgesia-fentanyl dose and breastfeeding success: a randomized clinical trial. Anesthesiology. 2017;127:614–24. 28926440 10.1097/ALN.0000000000001793

[6] Zhang T Yu Y Zhang W Zhu J. Comparison of dexmedetomidine and sufentanil as adjuvants to local anesthetic for epidural labor analgesia: a randomized controlled trial. Drug Des Devel Ther. 2019;13:1171–5. 10.2147/DDDT.S197431 PMC6469486 31043770

[7] Zhang L Xu C Li Y. Impact of epidural labor analgesia using sufentanil combined with low-concentration ropivacaine on maternal and neonatal outcomes: a retrospective cohort study. BMC Anesthesiol. 2021;21:229. 34551718 10.1186/s12871-021-01450-2 PMC8456635

[8] Liu L Drzymalski D Xu W Zhang W Wang L Xiao F. Dose dependent reduction in median effective concentration (EC50) of ropivacaine with adjuvant dexmedetomidine in labor epidural analgesia: an up-down sequential allocation study. J Clin Anesth. 2021;68:110115. 33142249 10.1016/j.jclinane.2020.110115

[9] Roelants F Rizzo M Lavand’homme P. The effect of epidural neostigmine combined with ropivacaine and sufentanil on neuraxial analgesia during labor. Anesth Analg. 2003;96:1161–6. 12651677 10.1213/01.ANE.0000050480.73209.9C

[10] Zhang W Li C. EC50 of epidural ropivacaine combined with dexmedetomidine for labor analgesia. Clin J Pain. 2018;34:950–3. 29595529 10.1097/AJP.0000000000000613

[11] Li G Wang H Qi X Huang X Li Y. Intrathecal dexmedetomidine improves epidural labor analgesia effects: a randomized controlled trial. J Int Med Res. 2021;49:300060521999534. 33827306 10.1177/0300060521999534 PMC8040578

[12] Li L Yang Z Zhang W. Epidural dexmedetomidine for prevention of intrapartum fever during labor analgesia: a randomized controlled trial. Pain Ther. 2021;10:391–400. 33188493 10.1007/s40122-020-00215-y PMC8119513

[13] Ngan Kee WD Ng FF Khaw KS Lee A Gin T. Determination and comparison of graded dose–response curves for epidural bupivacaine and ropivacaine for analgesia in laboring nulliparous women. Anesthesiology. 2010;113:445–53. 20613484 10.1097/ALN.0b013e3181bdf9da

[14] Bromage PR. A comparison of the hydrochloride and carbon dioxide salts of lidocaine and prilocaine in epidural analgesia. Acta Anaesthesiol Scand Suppl. 1965;16:55–69. 5322004 10.1111/j.1399-6576.1965.tb00523.x

[15] Xiang B Yang J Lei X Yu J. Adjuvant sufentanil decreased the EC50 of epidural ropivacaine for labor analgesia in healthy term pregnancy. Drug Des Devel Ther. 2021;15:2143–9. 10.2147/DDDT.S307478 PMC8140882 34040352

[16] Lee BB Ngan Kee WD Wong EL Liu JY. Dose–response study of epidural ropivacaine for labor analgesia. Anesthesiology. 2001;94:767–72. 11388526 10.1097/00000542-200105000-00013

[17] Duggan J Bowler GM McClure JH Wildsmith JA. Extradural block with bupivacaine: influence of dose, volume, concentration and patient characteristics. Br J Anaesth. 1988;61:324–31. 3179151 10.1093/bja/61.3.324

